# Approaches to improve Quality of Care (QoC) for women and newborns: conclusions, evidence gaps and research priorities

**DOI:** 10.1186/1742-4755-11-S2-S5

**Published:** 2014-09-04

**Authors:** Zulfiqar A Bhutta, Rehana A Salam, Zohra S Lassi, Anne Austin, Ana Langer

**Affiliations:** 1 Division of Women & Child Health, Aga Khan University, Karachi, Pakistan; 2 Program for Global Pediatric Research, Hospital For Sick Children, Toronto; 3 Harvard School of Public Health, Boston, USA

## Abstract

This series of papers focuses on a quality of care framework for maternal health, and systematically reviews the evidence of interventions aimed at improving care at the community-, district- and factility-levels. While the systematic reviews highlight the effectiveness of specific quality improvement efforts on maternal and newborn health, it also illlustrates the dearth of evidence on community-, district- and facility-level interventions, particulary for issues specific to quality of maternal health care and maternal newborn health outcomes. Further evidence is now needed to evaluate the best possible combination of the strategies. Governments, stakeholders and donors need to work together to form these policies and develop models of health care to suit the needs of their own population.

## Introduction

This series of papers focuses on a quality of care framework for maternal health, and systematically reviews the evidence of interventions aimed at improving care at the community-, district- and factility-levels. The approaches included in this paper had an effect on process and outcome measures for mothers and newborn health (MNH). Effects on other types of outcomes, while important, are not included in this analysis. In this final paper of the series, we summarize the most critical findings, and highlight the evidence gaps and research priorities identified through the analysis of the scientific literature. We also discuss the methodological quality of the existing evidence and areas for further advancement of the maternal and newborn health agenda, particularly in low and middle income countries (LMIC) [1-4].

## Overview of the findings

At the community-level, home visitation, community mobilization, women’s support groups and the training of community health workers (CHW) and traditional birth attendants (TBA) have shown significant and positive impacts on MNH outcomes. The community-based generation of funds for transportation also had an effect on access to MNH care in resource limited settings of India and sub-Saharan Africa. Mid-level health worker based care (MLHW) has not only demonstrated outcomes comparable to routine non-MLHW care delivery but also reported better results for some of the outcomes. Many of the interventions, including specialized outreach clinics, continuing medical education, problem-based learning, clinical practice guideline implementation and critical appraisal, showed inconclusive and mixed results on the quality of MNH care or MNH outcomes. Table 1 shows Key messages: Community-level

**Table 1:** Key messages: Community-level

• Packaged care involving outreach, referral, community mobilization and training have shown improvements in maternal and newborn health outcomes
• Midwife, TBA and CHW delivered care demonstrated significant improvements in maternal and newborn health outcomes

At the district-level, user directed financial incentives have shown to improve quality of care indicators, with conditional cash transfers and maternal voucher schemes having the most significant positive impacts across a range of MNH outcomes.

At the facility-level, evidence suggests that standardized or individualized social support programs and continuity of specialized midwifery care throughout pregnancy, labor and postnatal period have the potential to improve a range of perinatal, maternal, and labor specific indicators. To maintain performance and motivation among the healthcare workers, stress management trainings, multidisciplinary meetings and feedback sessions can reduce work related stress and improve performance. There was limited and inconclusive evidence for the impacts of physical environment, exit interviews and organizational culture modification on any MNH process or outcome measures. Table 2 shows Key messages: Disctrict-level.

**Table 2:** Key messages: District-level

• User directed financial strategies increase service utilization.
• Voucher schemes positively impact antenatal care, skilled attendance at birth, institutional deliveries and post-natal care.
• Supervision was found to positively influence provider’s practice, knowledge and awareness
• There is a dearth of evidence to conclude the effectiveness on district level inputs to improve maternal outcomes.

As discussed in paper 1 [1], this research was based on a review of systematic reviews of the evidence with some inherent methodological strengths and limitations. These should be considered while interpreting the findings of this series of papers.

## Key messages: Facility-level

• In-service training and specialty teams have conclusive benefits in improving maternal health outcomes.

• Social support during pregnancy interventions reduce antenatal hospitalization and caesarean delivery

• Strategies to improve professional practice were reported to have a significant positive effect on the desired practice.

## Evidence gaps and research priorities

While the systematic reviews highlight the effectiveness of specific quality improvement efforts on maternal and newborn health, it also illlustrates the dearth of evidence on community-, district- and facility-level interventions, particulary for issues specific to quality of maternal health care and MNH outcomes. This is particularly evident during the most hazardous time period for women (last trimester of pregnancy to the first week post-partum) when the majority of maternal deaths and severe morbidities occur.

Community-based quality improvement interventions were widely assessed for their effectiveness in improving MNH outcomes in low and middle income countries (LMIC). At the district-level, evidence from financial incentives was available from both high-income countries (HIC) and LMIC settings. Other interventions at the district-level were mainly evaluated in HIC settings. Given the differences in LMIC and HIC countries’ infrastructure and health systems, there is limited generalizability of findings across countries. There is also an information gap on the effectiveness of these interventions on different population groups that may represent within-country disparities.

At the district-level, many of the components of leadership, supervision, health information systems and staffing models had limited evidence of impact on the quality of maternal healthcare, or on MNH outcomes. Overall, very few maternal health specific outcomes were observed at the district-level. Although financial incentives, both user- and provider-directed, have been widely evaluated for their effectiveness in improving MNH outcomes, audits, feedbacks and information systems are mostly evaluated in the context of general health outcomes. Moreover, reviews focusing on MNH specific interventions, like maternal and perinatal mortality audits, require strong, standardized data collection mechanisms to evaluate their effectiveness. Improving the health information systems in countries is necessary for any evaluation of the impact of mortality audits.

From the facility-level evidence, most of the findings from social support and specialized midwifery teams programs during pregnancy and labor were limited to HIC. There is limited evidence on the effects of these interventions on maternal and newborn health outcomes in LMIC. There is also a lack of data evaluating the effectiveness of structural and cultural changes, educational interventions, and the facility mix of staff skills. Future research on the impact of these interventions (at the facility-level) on quality of maternal health care and specific maternal health outcomes in LMIC’s is essential in order to make evidence-based recommendations for better policies, programs and practice.

Very few of these studies provided evidence on sustainability and scale up, particularly in countries where resources are constrained and health systems are weak. Recent studies in Uganda and Ghana have highlighted the challenges in scaling up interventions that have been proven effective [5,6]. Interventions that have been proven to improve MNH outcomes merit further research. Research should focus on the factors affecting the sustainability of these interventions when scaled up, and the cost-effectiveness of these interventions. It is important to understand if quality improvement interventions are associated with overall health care savings. Another area for future research is evaluating how the highest impact interventions to address quality of care can be implemented in a variety of contexts and settings [7].

There are currently several innovative interventions that are being implemented globally to improve the quality of maternal health services. The Maternal Health Task Force, for example, is currently supporting multiple research projects aimed to improve referral systems, increase the use of novel methodologies to train providers, introduce innovative approaches to supportive supervision and mentoring, increase access to blood products, develop professional organizations, among many others. The results of the evaluation of the impact of these programs on the quality of maternal care may fill many of the informational gaps in the Quality of Maternal Care Framework that this systematic review has utilized.

Finally, qualitative data describing individual components of the interventions for reproducibility will be invaluable for scale up and sustainability. Further evidence is now needed to evaluate the best possible combination of the strategies. Governments, stakeholders and donors need to work together to form these policies and develop models of health care to suit the needs of their own population. This will further lead to outlining approaches that enable health care providers either in the community or in a facility, and program managers at the district-level, to adopt and implement patient-centered, evidence-based interventions to improve the quality of care during childbirth and the immediate postpartum period.

Research and programming priorities are summarised in Table 3.

**Table 3:** Key messages - Facility level

• Much of the data has been collected in high- resource settings. More evidence from low-resource settings needs to be generated.
• Regional and urban/rural discrepancies within countries need to be examined.
• More interventions need to be evaluated using quality maternal health indicators as an outcome.
• Indicators of quality care need to be standardized to facilitate the evaluation of quality improvement efforts
• Strengthening health information systems is required to evaluate many interventions’ effectiveness.
• Evidence on the sustainability of proven interventions should be generated, including evidence on feasibility of implementation and scale up in a variety of settings.
• Current efforts to improve quality of care and maternal and newborn health outcomes in developing countries should have a strong evaluation component to contribute to the evidence base.
• Mixed method approaches to evaluation would add depth to the evidence and would uncover hidden barriers and supporting factors for implementation and scale up of best practices.

## Competing interests

We do not have any financial or non-financial competing interests for this review.

## Authors' contribution

All authors contributed in writing the paper.

## Supplementary Material

Additional file 1 Click here for file

## Declarations

This article has been published as part of *Reproductive Health* Volume 11 Supplement 2, 2014: Quality of Care in Maternal and Child Health. The full contents of the supplement are available online at http://www.reproductive-health-journal.com/supplements/11/S2.
