# Supplementary material for: Approaches to improve Quality of Care (QoC) for women and newborns: conclusions, evidence gaps and research priorities
Source: Reprod Health. 2014 Sep 4;11(Suppl 2):S5. doi: 10.1186/1742-4755-11-S2-S5 (PMC4160923; doi:10.1186/1742-4755-11-S2-S5)
Supplement: Additional file 1 [file 1742-4755-11-S2-S5-S1.pdf]

Referee's comments to the authors– this sheet WILL be seen by the author(s) and published alongside the article

Article ref no.	
Title	Evidence from community level inputs to improve quality maternal and newborn health: Interventions and findings
Author(s)	Zohra S lassi; Jai K. Das; Rehana A Salam & Zulfiqar A Bhutta
Referee's name	J. Shea

When assessing the work, please consider the following points, where applicable:

[USE THE APPROPRIATE QUESTIONS FOR THE ARTICLE TYPE TO BE REVIEWED – SEE Reviewer Guidelines above]

1. Is the question posed by the authors new and well defined?
2. Are the methods appropriate and well described, and are sufficient details provided to replicate the work?
3. Are the data sound and well controlled?
4. Does the manuscript adhere to the relevant standards for reporting and data deposition?
5. Are the discussion and conclusions well balanced and adequately supported by the data?
6. Do the title and abstract accurately convey what has been found?
7. Is the writing acceptable?

Please make your report as constructive and detailed as possible in your comments so that authors have the opportunity to overcome any serious deficiencies that you find and please also divide your comments into the following categories:

- Major Compulsory Revisions (which the author must respond to before a decision on publication can be reached)
- Minor Essential Revisions (such as missing labels on figures, or the wrong use of a term, which the author can be trusted to correct)
- **Discretionary Revisions (which are recommendations for improvement but which the author can choose to ignore)**

Where possible please supply references to substantiate your comments.

When referring to the manuscript please provide specific page and paragraph citations where appropriate.

This final paper is a good summation of the series.
The comment on leadership/supervision is not a fair reflection of the content since leadership was not adequately discussed. Moreover, leadership and supervision, though interrelated, are not the same thing.

Language/editing issues
- and systematically reveiws (p.2) – reviews
- mothers and newborns health (MNH) (p.2) – newborn
- To maintain performance and motivation among the healthcare workers (p.2) – delete the unless using the health workforce
- While the systematic reviews hilight the effectiveness of specific quality improvement efforts on maternal, newborn health (p.3) – maternal and newborn health

- where the majority of maternal deaths and severe morbidities occur (p.3) – when
- Community based quality improvement interventions were most widely assessed (p.3) – why most widely?
- There is also an information gap on effectiveness (p.3) – insert the
- programs during pregnancy and labor were limited to the developed countries (p.3) – delete the
- There is also lack of data evaluating the effectiveness (p.3) – insert a
- at the commuity, district and facility level on quality of matenal health care (p.3) – correct spelling
- Very few of these studies, provided evidence on sustainability and scale up (p.4) – delete the comma
- Several recent studies in Uganda and Ghana have highlighted the challenges in scaling up interventions that have been proven effective (p.4) – revise
- Interventions that have proven to improve MNH outcomes merit further research on the factors affecting the sustainability of these interventions when scaled up; and the cost-effectiveness (p.4) – revise
- It is important to define if interventions to improve quality of care are associated with overall health care savings (p.4) - revise.
- Another area for further research is evaluating how the best high-impact interventions to address quality of care can be implemented in a variety of contexts and settings.(7)(p.4) - revise
- research projects aimed to improve referral systems (p.4) – at
- Further evidence is now needed to evaluate the best possible combination of the strategies (p.4) – revise
- This will further lead to outlining approaches that enable health care providers either in the community or in a facility, and program managers at the district level, to adopt and implement patient-centered, evidence-based interventions to improve the quality of care during childbirth and the immediate postpartum period – consider revising

Althabe: reviewer comments for paper 5:

Nice manuscript, well summarized and written. Difficult to review without reviewing paper series 2-4. However doesn't seem to be major issues. I attach the manuscript with minor spelling errors.

Thank you for noting the spelling errors, which have been corrected.

Shea: reviewer comments for paper 5:

This final paper is a good summation of the series.

The comment on leadership/supervision is not a fair reflection of the content since leadership was not adequately discussed. Moreover, leadership and supervision, though interrelated, are not the same thing.

Thank you for pointing this very important difference between leadership and supervision. Please see the expanded definition of leadership in paper 3. We have differentiated between leadership and supervision in paper 5.

Language/editing issues

- and systematically reveiws (p.2) – reviews
- mothers and newborns health (MNH) (p.2) – newborn
- To maintain performance and motivation among the healthcare workers (p.2) – delete the unless using the health workforce
- While the systematic reviews hilight the effectiveness of specific quality improvement efforts on maternal, newborn health (p.3) – maternal and newborn health
- where the majority of maternal deaths and severe morbidities occur (p.3) – when
- Community based quality improvement interventions were most widely assessed (p.3) – why most widely?
- There is also an information gap on effectiveness (p.3) – insert the
- programs during pregnancy and labor were limited to the developed countries (p.3) – delete the
- There is also lack of data evaluating the effectiveness (p.3) – insert a
- at the commuity, district and facility level on quality of matenal health care (p.3) – correct spelling
- Very few of these studies, provided evidence on sustainability and scale up (p.4) – delete the comma
- Several recent studies in Uganda and Ghana have highlighted the challenges in scaling up interventions that have been proven effective (p.4) – revise
- Interventions that have proven to improve MNH outcomes merit further research on the factors affecting the sustainability of these interventions when scaled up; and the cost-effectiveness (p.4) – revise

- It is important to define if interventions to improve quality of care are associated with overall health care savings (p.4) - revise.
- Another area for further research is evaluating how the best high-impact interventions to address quality of care can be implemented in a variety of contexts and settings.(7)(p.4) - revise
- research projects aimed to improve referral systems (p.4) – at
- Further evidence is now needed to evaluate the best possible combination of the strategies (p.4) – revise
- This will further lead to outlining approaches that enable health care providers either in the community or in a facility, and program managers at the district level, to adopt and implement patient-centered, evidence-based interventions to improve the quality of care during childbirth and the immediate postpartum period – consider revising

Thank you for your excellent editing finds-We have made all revisions suggested.

Prof Jos van Roosmalen : Response to reviewer

Paper 5. Quality of care for maternal health: priorities for research and action

I remain with a strange feeling, that these papers are a systematic review of systematic reviews and may be not as systematic as such a review should be. Not all relevant papers appear in systematic reviews as I showed already before. Also ignoring papers (as I mentioned before) is strange as the review recommends to perform the type of studies which already have been performed in recent years.

Thanks. We have now mentioned the methodological strengths and limitations of overview of systematic reviews in Paper 1 and also cautioned the readers to consider the same when interpreting the findings.

Another problem is that the issue of continuous support is researched mainly in HIC and that the conclusion then is that there is no eveidence for LMIC! I do not agree, as Hodnett's Cochrane explicitly state that their findings are especially relevant where there is no pain relief during labour.

Hodnett's review was incorporated into paper two, at the community level- as it evaluated interventions delivered during home visits, during regular antenatal clinic visits, and/or by telephone on several occasions during pregnancy. We note that community-based evidence for quality improvement interventions are available from both HIC and LMIC (page 3) Thank you.

We do not need trials to create “evidence” that continuous support would work in LMIC, where most women deliver alone in a room with other laboring women with a lot of anxiety, pain and loneliness. The paper should therefore ask the important question why do most women in LMIC have to deliver their babies without continuous support, while this is a low tech strategy which could be implemented without any cost and would certainly in analogy of what we already know, have great positive impact on maternity care.

We fully agree with your points. Receiving support during pregnancy and delivery is a basic human right. Several groups in the maternal health community are working on developing indicators and standards to measure disrespect and abuse, and designing, implementing and evaluating different approaches to improve respectful maternal care. We need a better understanding of “how” to implement what we know works.

We added a call for research, which we state on page 5.

The authors have to address these issues, will these papers have any impact.

The most impertinent statement in paper 5 is: “Since there is no evidence from LMIC countries on the impact of such interventions (i.e. continuous support), cross-country generalizability is not possible”. This cannot be left in the paper, and instead the question should be addressed why is a cost-effective, low cost intervention not implemented in LMIC. We really do not need more evidence than we already know from all those labour wards where women are dehumanized and have to deliver their babies in isolation without any pain relief.

We modified the sentence you found offensive. The dearth of evidence from LMIC we referred to was at the facility level. While support during labor is an ethical imperative, the evidence on its impact on maternal and newborn health in LIMIC is, indeed, limited.
